# Quantifying Plant Signaling Pathways by Integrating Luminescence-Based Biosensors and Mathematical Modeling

**DOI:** 10.3390/bios14080378

**Published:** 2024-08-05

**Authors:** Shakeel Ahmed, Syed Muhammad Zaigham Abbas Naqvi, Fida Hussain, Muhammad Awais, Yongzhe Ren, Junfeng Wu, Hao Zhang, Yiheng Zang, Jiandong Hu

**Affiliations:** 1 College of Mechanical and Electrical Engineering, Henan Agricultural University, Zhengzhou 450002, China; shakeeel.ahmed@stu.henau.edu.cn (S.A.); zaigham@stu.henau.edu.cn (S.M.Z.A.N.); fidahussain@stu.henau.edu.cn (F.H.); dr.muhammad.awais@henau.edu.cn (M.A.); jfwu@henau.edu.cn (J.W.); hao.zhang@henau.edu.cn (H.Z.); yheng@stu.henau.edu.cn (Y.Z.); 2 Henan International Joint Laboratory of Laser Technology in Agriculture Sciences, Zhengzhou 450002, China; 3 State Key Laboratory of Wheat and Maize Crop Science, Zhengzhou 450002, China; yzren@henau.edu.cn

**Keywords:** plant hormone signaling pathways, abscisic acid, genetically engineered bacteria, bioluminescence, mathematical modeling, simulations

## Abstract

Plants have evolved intricate signaling pathways, which operate as networks governed by feedback to deal with stressors. Nevertheless, the sophisticated molecular mechanisms underlying these routes still need to be comprehended, and experimental validation poses significant challenges and expenses. Consequently, computational hypothesis evaluation gains prominence in understanding plant signaling dynamics. Biosensors are genetically modified to emit light when exposed to a particular hormone, such as abscisic acid (ABA), enabling quantification. We developed computational models to simulate the relationship between ABA concentrations and bioluminescent sensors utilizing the Hill equation and ordinary differential equations (ODEs), aiding better hypothesis development regarding plant signaling. Based on simulation results, the luminescence intensity was recorded for a concentration of 47.646 RLUs for 1.5 μmol, given the specified parameters and model assumptions. This method enhances our understanding of plant signaling pathways at the cellular level, offering significant benefits to the scientific community in a cost-effective manner. The alignment of these computational predictions with experimental results emphasizes the robustness of our approach, providing a cost-effective means to validate mathematical models empirically. The research intended to correlate the bioluminescence of biosensors with plant signaling and its mathematical models for quantified detection of specific plant hormone ABA.

## 1. Introduction

Plant cells have intricate signaling pathways enabling them to perceive and respond to environmental changes through gene expression and regulatory mechanisms [1,2]. When plant cells detect stress signals, a series of events is triggered, leading to the activation of genes and the production of important secondary messengers, such as phytohormones, reactive oxygen species (ROS), and calcium ions (Ca^2+^) [3]. These messengers drive physiological adaptations and systemic signal transduction, enabling plants to acclimate. Studies have shown that plants can integrate different local and systemic signals generated during conditions of stress combination [4]. During abiotic stress, the most well-studied communication system is the long-distance communication between plant organs by gibberellins (GAs), abscisic acid (ABA), and cytokinins, auxins [5]. Abscisic acid controls various plant biological processes, including seed dormancy, germination, stomatal movement, floral induction, and leaf senescence. It also plays a crucial role in regulating plant reactions to different environmental pressures, including drought, salinity, cold, heat, and pathogen invasion. ABA operates by regulating the expression of genes that respond to stress, generating secondary messengers, and initiating signaling pathways to trigger plant stress responses [6,7,8]. Plant developmental biology faces the challenge of understanding how genes and hormones interact to coordinate growth amid changing environments [9].

Mathematical modeling is essential in deciphering how plant hormones govern cell fate and behavior. These models reveal the dynamics and underlying mechanisms of hormone signaling pathways by integrating experimental data and testing hypotheses. They predict outcomes under various scenarios and perturbations, offering a quantitative framework to analyze complex interactions among receptors, kinases, transcription factors, and other molecules [10,11]. However, their effectiveness hinges on the availability of precise experimental data. Advances in sensing technologies, omics approaches, and quantitative microscopy are vital for providing detailed, spatiotemporal data on metabolites, gene expression, protein activity, and network connectivity, thereby enhancing modeling strategies [12]. Various approaches have been employed to model plant cell signaling, including Boolean network models, partial differential equation (PDE)-based models, stochastic models, agent-based models, network modeling techniques, and ordinary differential equation (ODE)-based models. Boolean network models represent ABA signaling as discrete states [13,14], while PDE-based models describe the spatial distribution of signaling molecules in tissues/organs [15]. Stochastic models capture the probabilistic nature of ABA signaling events [16], and agent-based models simulate individual plant cells and their interactions [17]. Network modeling techniques help analyze and understand complex interactions within signaling pathways and networks in plant cells [18], whereas ODE-based models incorporate critical components of the ABA signaling pathway [19,20,21]. These modeling approaches provide insights into plant signaling systems’ regulatory mechanisms, signal transduction processes, and emergent behaviors.

**Figure 1:**
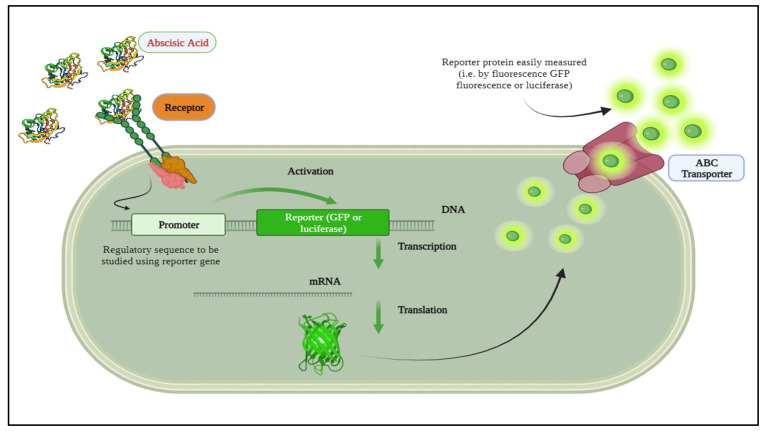
A whole-cell biosensor integrated with bioluminescence.

Recent advancements in biotechnology, biochemistry, and bioengineering have made it possible to quantify information molecules conveyed among plant cells. A biosensor is an analytical instrument that combines a biological recognition component with a physical transducer to produce a quantifiable signal directly proportional to the concentration of the substances being analyzed [22,23]. Reporter proteins, such as those measured by GFP fluorescence (excitation/emission 488/533 nm) [24] or luciferase “hν~490 nm” [25], exemplify this progress. For instance, researchers have developed a quantitative autonomous bioluminescence reporter system with a wide dynamic range, utilizing the bioluminescence pathway from Neonothopanus Nambi, eliminating the need for external luciferase substrates and making it cost-effective and suitable for high-throughput applications [26]. Additionally, ABA biosensor detection capabilities have been enhanced through genetic screening techniques using luciferase reporters driven by stress-responsive promoters [27]. Evaluations of different reporter systems, including GUS, LUC, and GFP, emphasize the importance of selecting appropriate reporter systems for specific applications to develop efficient ABA biosensors [28]. These advancements underscore the potential of these technologies in improving ABA detection and enhancing plant studies. Accurate detection and quantification of ABA are essential for understanding physiological processes and improving agricultural practices. In this regard, luminescent biosensors offer the potential for real-time rapid, sensitive, and particular ABA detection [29,30]. By integrating bioluminescence with gene expression systems, whole-cell biosensors can monitor intracellular ABA levels in real-time scenarios [31], as depicted in Figure 1.

Identifying and observing the light emitted by bioluminescent species, such as bioluminescent sensors, is possible through bioluminescence microscopy. This method entails using a highly responsive camera, usually equipped with a specialized detector like a photomultiplier tube, to catch the light emitted by the bioluminescent sensor. The camera captures the emitted light, enabling researchers to observe and analyze the bioluminescent activity of the sensor [32,33]. Bioluminescence offers significantly greater quantum yields compared to classical chemiluminescence [34]. Various modifications to luciferin and luciferase enzyme mutations enable the development of customizable biosensors with tunable emission of different colors like green, yellow, and even blue light, with different wavelengths [35,36,37]. Genetically engineered with luciferase genes fused to target-activated promoters, live cells produce a luminescent signal in response to specific compounds or stress conditions, often obviating the need for external substrates when using bacterial luciferase due to the co-transcription of substrate-generating genes [38,39,40]. An ideal biosensor used for quantitative analysis should exhibit a wide range of biologically substantial sensitivity, have minimal impact on the system it is used in, be easily detectable, possess a high signal-to-noise ratio, and provide either relative or absolute quantification of the targeted signaling event or substrate under investigation [41,42].

The intricate nature of plant signaling pathways and the pragmatic difficulties linked to experimentally replicating all potential stress factors on live plants have led to a transition toward simulation methodologies. Performing comprehensive experiments is not only laborious but can also be cost-prohibitive. Hence, it is imperative to integrate simulation approaches into our research. Using simulations, we create a novel opportunity to investigate plant hormone signaling pathways more efficiently and cost-effectively. Simulation methods are crucial in enhancing experimental data by offering a quantitative framework for analyzing and replicating the complex dynamics of these pathways. Our research in this context utilizes simulation techniques as a valuable tool to improve our comprehension of how plants respond to stimuli and contribute to the progress of this subject.

## 2. Material and Methods

### 2.1. Simulating ABA Interaction with PP2C/SnRK2/MAPK

**Figure 2:**
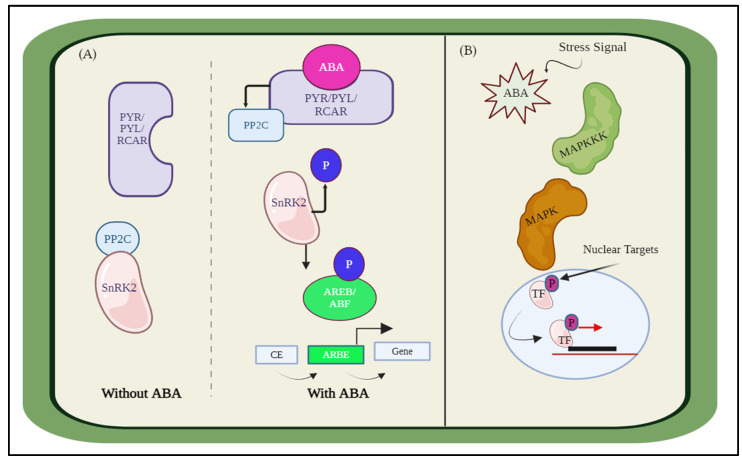
Abscisic acid (ABA) interaction with other hormones (**A**) ABA response for abiotic stress response, stomatal closure, seed germination, root development, etc. (**B**) Interaction of ABA with MAPK.

We presented the modeling of plant essential hormones employing the Hill equation and the ODEs. First, we discuss the biological perspective of hormonal interaction and then model this interaction. The core signaling pathways that are responsible for ABA receptor coupling are made up of three main components: the ABA receptors, the PYRABACTIN RESISTANCE (PYR)/PYR-LIKE (PYL), also known as REGULATORY COMPONENTS OF ABA RECEPTORS (RCAR) family proteins, the negative regulator clade A type 2 C protein phosphatases (PP2Cs), and the positive regulator SNF1-related protein kinase 2s (SnRK2s). Therefore, the activation of SnRK2s and ABA signaling is determined by phosphorylation and dephosphorylation [43], as shown in Figure 2A. Further, the interplay between ABA signaling and other signaling pathways, such as the mitogen-activated protein kinase (MAPK) pathways, Figure 2B, which are essential for stress responses and plant growth and development, is another critical feature of ABA signaling. Several MAPK signaling components, including MPK3, MPK6, and MKK9, exhibit transcriptional modulation, protein accumulation and stability, and kinase activity upon receiving ABA therapy. The expression and function of ABA-responsive genes and proteins, including transcription factors, ion channels, and enzymes, are modulated by these MAPKs [44]. These interactions have been simulated for visualization and discussed in the results.

We have developed the code that simulates the interaction of proteins SNRK2, PP2C, and MAPK with the plant hormone abscisic acid (ABA). The parameters and methodology are based on the following references:

i. The Hill equation is a widely used model to describe the binding of ligands to receptors or enzymes. The parameters “*K*” (half-maximal concentration) and “n” (Hill coefficient) are commonly used in this equation [45,46].
ii. The rate constants “*K*_1_”, “*K*_2_”, and “*K*_3_” are typical parameters used in ODE models [19,20,47] for chemical reactions and biological processes [48]. The constants “*K*_1_”, “*K*_2_”, and “*K*_3_” describe critical processes within our model. “*K*_1_” represents the rate constant for ABA production, “*K*_2_” accounts for the degradation of ABA, and “*K*_3_” describes the rate of ABA binding to and dissociating from its receptor. These constants are crucial for accurately modeling the dynamic behavior of ABA within the plant system.
iii. The interaction constants “*k*_interact_SNRK2_”, “*k*_interact_PP2C_”, and “*k*_interact_MAPK_” represent the interaction rates between the respective proteins. These are often determined experimentally or estimated based on similar systems [49].
iv. The initial concentrations (“*C_ABA_0_*”, “*SNRK2_0*”, “*PP2C_0*”, “*MAPK_0*”) and the time span (“tspan”) are specific to the system being modeled and can be adjusted as needed.

#### 2.1.1. Protein Interaction with ABA

Defined parameters and variables for simulating protein–protein interaction and the Hill equation that models the cooperative binding of ABA to its receptor are given as:

(1) Hill equatin=(CABA)n(Khalf min,max)n+(CABA)n

where $C_{ABA}$, is ABA concentration.

The system of ordinary differential equations (ODEs) for protein–protein interactions with ABA is defined as:

(2) dCABAdt=ksynthesis−K1·CABA

(3) dSNRK2dt=K2·Hill Equation

(4) dPP2Cdt=kinteractSNRK2·SNRK2−kintericatPP2C·PP2C

(5) dMAPKdt=−kinteractSNRK2·SNRK2+kinteractMAPK·PP2C+K3·ABA

These equations model the dynamics of ABA concentration $C_{ABA}$, *SNRK2*, *PP2C*, and *MAPK* interactions over time.

#### 2.1.2. Protein Interaction without ABA

This code simulates the interaction of proteins SNRK2, PP2C, and MAPK without the involvement of ABA. The parameters and methodology are similar to the first code, with the following differences: The ABA-related terms (“*k_synthesis_*”, “*K*_1_”, and the Hill equation) are removed since ABA is not considered in this simulation. The ODE system is simplified to include only the interactions between the three proteins, and the initial conditions and time span are specific to this simulation without ABA.

For interactions without ABA, the system of ODEs is simplified to focus only on protein–protein interactions:

(6) dPP2Cdt=kinteractSNRK2·SNRK2−kintericatPP2C·PP2C

(7) dMAPKdt=−kinteractSNRK2·SNRK2+kinteractMAPK·PP2C

(8) dSNRK2dt=−kinteractMAPK·PP2C

### 2.2. Integrating Biological Sensors with Mathematical Modeling of Plant Signaling for Improved Understanding of Plant Signaling

Integrating luminescent biosensors with mathematical modeling to quantify plant signaling pathways offers a robust approach to enhancing our understanding of plant responses and our ability to monitor and manipulate them. This integration provides several potential benefits [12]. Identifying and observing the light emitted by bioluminescent species is possible through bioluminescence microscopy [32]. Biological sensors combined with mathematical models enable real-time monitoring of plant responses [50], quantitative analysis of the underlying signaling mechanisms [50,51], prediction of plant responses under different conditions, and system optimization of plant responses [12,52,53]. Autonomous bioluminescence reporter system and genetic screening techniques were described earlier [26,27].

#### Establishing the Relationship between ABA Signaling, Mathematical Model, and Biological Sensor

The development of the autonomous bioluminescence reporter system by [26] is an example of a recent breakthrough in plant synthetic biology. This system offers a reliable method for quantifying gene expression. This system utilizes the bioluminescence pathway from Neonothopanus nambi, eliminating the requirement for external luciferase substrates. As a result, it is both cost-effective and capable of handling many samples simultaneously. In addition, the genetic screening procedures outlined by [27] utilizing luciferase reporters controlled by stress-responsive promoters significantly improves ABA biosensors’ detection capacities. The Hill equation has been applied to establish a mathematical model that correlates ABA signaling and bioluminescent sensors responding with bioluminescence [45,46]. It is commonly utilized in pharmacology and biochemistry, especially for capturing cooperative binding or activation processes. The association rate constant $k_{on}$ represents the rate at which ABA molecules bind to the biosensor receptors, measured in M^−1^ s^−1^ (moles per liter per second). This constant is crucial for understanding the biosensors’ binding kinetics and efficiency *n*, detecting ABA. For various biological interactions, $k_{on}$ values can differ significantly. For instance, TCR–pepMHC interactions typically have $k_{on}$ values ranging from 10^3^ to 10^5^ M^−1^ s^−1^ [54,55]. In antibody–antigen interactions, the association rate constants are generally higher, falling between 10^5^ and 10^6^ M^−1^ s^−1^. For a reasonable range for *k_on_* for ABA biosensors 10^4^ to 10^6^ M^−1^ s^−1^, reflecting the rapid binding required for efficient real-time detection of ABA levels in plant physiological studies. Higher $k_{on}$ values ensure rapid binding of ABA to the biosensor, making the sensor responsive and efficient for real-time detection.

Similarly, the dissociation rate constant $k_{off}$ represents the rate at which ABA molecules dissociate from the biosensor receptors, measured in s^−1^ (per second). Typical $k_{off}$ value varies depending on the type of interaction. For TCR–pepMHC interactions, dissociation half-times ranges from a few seconds to 1–2 min. In antibody–antigen interactions, dissociation half-times can range from a few minutes to an hour or more. FA reasonable range for *k_off_* for ABA biosensors 10^−2^ to 10^−4^ s^−1^ [54,55], ensuring the ABA–receptor complex remains stable for adequate signal measurement. Lower $k_{off}\;$ value ensures the ABA–receptor complex remains stable for adequate signal measurement.

To integrate innovative bioluminescence systems [26,27] with our mathematical model, we define the model that incorporates the terms for synthesizing and degrading sensor protein mRNA and the sensor protein itself, along with the interaction between ABA and the sensor protein. Defining the gene/protein expression and interaction (sensor protein) dynamics, we modeled the transcription of sensor protein mRNA as

(9) d(mRNA)dt=ktrans·[(CABA)n(khalf)n+(CABA)n]−ktran_deg·(mRNA)

and translation of sensor protein as

(10) d(Protien)dt=ktran_syn·(mRNA)−kdeg_port·(Protein)

The interaction between *ABA* and its receptors as association and dissociation of *ABA* take into account as synthesized sensor protein,

(11) $$ABA+Protein\;\begin{array}{c} k_{on}\\ ⇌\\ k_{off} \end{array}\;ABA\cdot \;Protein$$

The bioluminescence response is established considering the Hill equation,

(12) IBioluminescence=(CABA·Protein)n[Khalf n+(CABA·Protein)n]

Combining the above equations, we obtain the following system of ODEs, for *ABA* concentration dynamics:

(13) d(CABA)dt=kprod−kdeg·CABA−kon·(CABA·Protein)+koff·(CABA·Protein)

for *ABA*—receptor complex dynamics:

(14) d(CABA·Protein)dt=kon·(CABA·Protein)−koff·(CABA·Protein)

and for the rate of change in bioluminescence:

(15) d(IBioluminescence)dt=kresponse·(CABA·Protein)n[Khalf n+(CABA·Protein)n]

The initial *ABA* concentration varies from 1.5 to 50 µmol, whereas the values for $C_{ABA}\cdot Protein$, mRNA, Protein, and $I_{Bioluminescence}$ sets to zero in simulations.

This model incorporates detailed gene/protein expression terms, promoter rate constants, and enzyme kinetics, providing a more accurate representation of the ABA biosensor system. The MATLAB simulation results demonstrate the relationship between initial ABA concentration and the final bioluminescence response, illustrating the proportional relationship between detected enzyme activity and bioluminescence.

## 3. Results

### 3.1. Visualizing the Interaction of SnRK2, PP2C and MAPK with ABA

**Figure 3:**
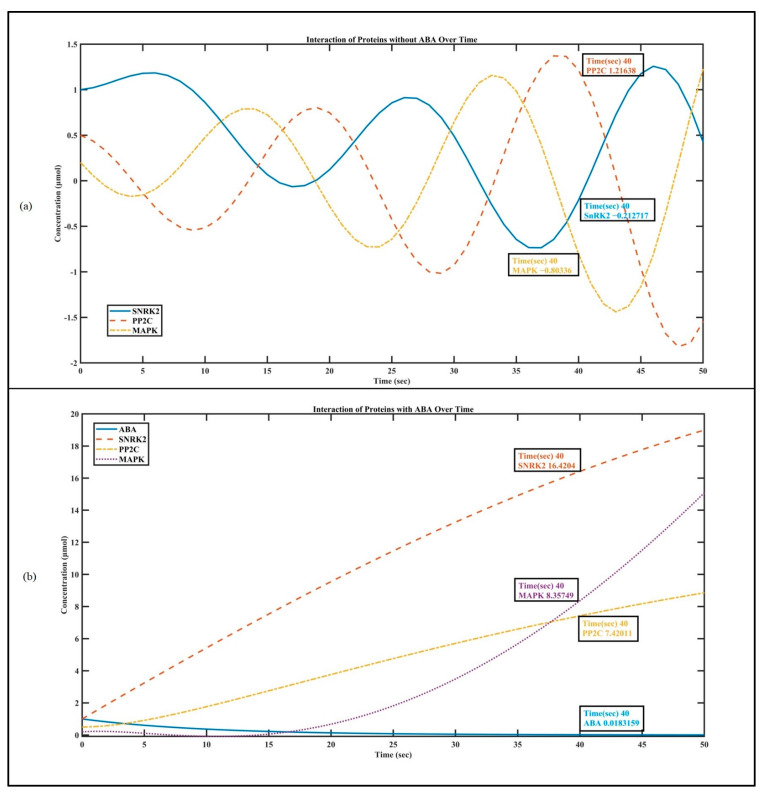
Plant hormone interaction: (**a**) interaction of SnRK2, PP2C, and MAPK without ABA; and (**b**) interaction of SNRK2, PP2C, and MAPK in the presence of ABA.

In the first part, we simulate the interaction of SnRK2, PP2C, and MAPK with ABA using ODEs [19,21] and associate it with the Hill equation [45,46]. The related ODEs and the Hill equation with these proteins model their rates of change over time, capturing the dynamic nature of their concentrations in response to the absence and presence of ABA. Interaction constants (*k_interact_SNRK2_*, *k_interact_PP2C_*, and *k_interact_MAPK_*) are introduced to further simulate the interaction between proteins. The code generates visualizations that enable the observation of how these protein interactions evolve over time. The plot illustrating protein concentrations provides a dynamic snapshot of the intricate ballet between SnRK2, PP2C, and MAPK in Figure 3. Without ABA, Figure 3a shows that PP2C becomes the primary focus. It deactivates SnRK2 by directly removing its phosphate groups. This inhibition hinders SnRK2 from phosphorylating subsequent transcription factors, slowing down ABA signaling [55].

When environmental or developmental cues trigger the presence of ABA Figure 3b, a captivating transformation occurs. ABA binds to PYR/PYL/RCAR proteins, forming a complex. This complex then interacts with PP2C, leading to PP2C inhibition. As a result, SnRK2 awakens from its slumber, ready to activate a cascade of ABA responses [55].

### 3.2. Simulating the Relationship between ABA Signaling and Bioluminescent Sensors (ABA-Luminescence Model)

**Figure 4:**
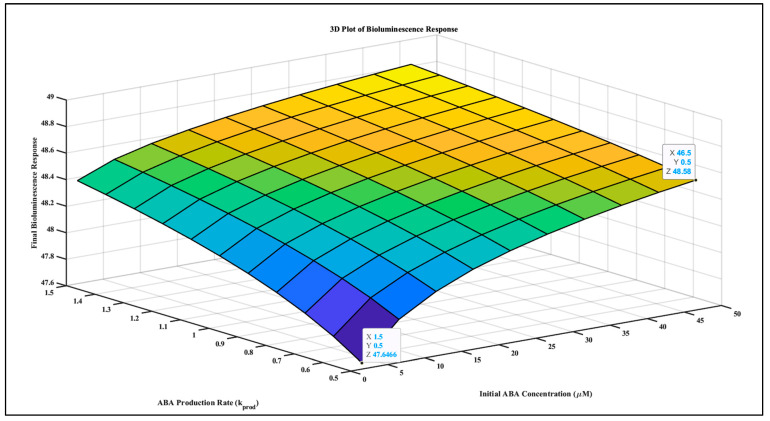
The plot of ABA concentration (µmol) and bioluminescence (RLUs) shows proportionality between varying ABA concentration and resulting bioluminescence. The gradient from blue to orange on the plot represents bioluminescence intensity. Blue color is indicating the lower and orange color is indicating the higher levels, corresponding to ABA concentrations and production rates.

A simulation was created to study the relationship between ABA signaling and the bioluminescence of biosensors. MATLAB algorithm using the Hill equation in a system of ODEs was used to capture the interaction between ABA signaling and the sensor response. Bioluminescence intensity is integrated cumulatively based on the sensor’s response. Figure 4 shows the correlation between ABA concentrations and bioluminescence intensity, which varies based on the system’s biological response.

## 4. Discussions

This article discusses the theoretical analysis of plant signaling pathways, the role of mathematical modeling in studying these pathways, ABA signaling, and the effectiveness of bioluminescent sensors. Utilizing the property of bioluminescence sensors for testing mathematical models of plant signaling pathways is valuable, leading to the development of optical biosensors. Given the complexity of a plant’s signaling pathways and the practical difficulties associated with replicating all potential stress factors on live plants, researchers increasingly turn to simulation methodologies for rapid results, avoiding laborious experimental procedures and high costs. This discussion emphasizes plant signaling, particularly ABA, the advantages of bioluminescent sensors, and mathematical models used to model plant signaling.

Existing models in the literature have also explored the interactions between ABA and various proteins. A study investigated the detailed mechanisms of ABA signaling, focusing on the interactions between ABA, PP2C, and SNRK2 [55]. Whereas [56] detailed the rate constants and reaction mechanisms derived from experimental data. Another study by [57] explored the phosphorylation–dephosphorylation cycles involving ABA, PP2C, and SNRK2. Our model builds upon these foundational studies by incorporating a simplified yet comprehensive approach to simulate the dynamic interactions between ABA and proteins. Using the Hill equation to describe cooperative binding and employing ordinary differential equations, our model captures the essential dynamics of these interactions. This approach allows for flexible parameter adjustments, providing insights into the system’s behavior under different conditions.

Calvache et al. developed the Quantitative Autonomous Bioluminescence Reporter System [26], designed to operate without external luciferase substrates, emphasizing its wide dynamic range and high sensitivity across varying target molecule concentrations. Chinnusamy et al. [27] discuss Screening for Gene Regulation Mutants by Bioluminescence Imaging utilizing the RD29A::LUC construct in Arabidopsis, demonstrating that bioluminescence driven by the RD29A promoter correlates directly with ABA concentration and other stress factors. Our mathematical model incorporates the Hill equation and ordinary differential equations (ODEs) to depict ABA–receptor binding dynamics, biosensor transcription and translation, and resultant bioluminescence. Parameters such as association and dissociation rates, synthesis and degradation rates, and the Hill coefficient define the biosensor’s sensitivity and response curve. Our model’s initial bioluminescence of 47.646 RLUs for 1.5 μmol of ABA underscores its high sensitivity, aligning with the sensitivity and dynamic range requirements emphasized by [26,27]. The model demonstrates a linear relationship between initial ABA concentrations and bioluminescence.

## 5. Conclusions

This study demonstrates the effective use of computational models to simulate plant signaling pathways, specifically focusing on ABA interactions and bioluminescence as a quantifiable response. Taking advantage of the bioluminescence emission’s proportionality to the identified enzyme makes it a valuable tool for testing mathematical models of plant signaling pathways, leading to the development of optical biosensors. The complexity of plant signaling pathways, the practical difficulties in replicating all potential stress factors on live plants, and the intricacy of biological processes increasingly lead researchers to utilize simulation methodologies for rapid results, avoiding laborious experimental procedures and high costs. The simulated integration of ABA with bacterial luminescence using the ODE model revealed that modified bacteria can respond to varying concentrations of ABA from 1.5 to 46.5 µM by creating 47.6466 to 48.8 RLUs of bioluminescence. Based on our simulated model, we predict that combining these approaches allows researchers to understand plant signaling better and gain new insights into plant growth, development, and stress responses. Furthermore, the performed sensitivity analysis provides a way forward for experimental design and parameter estimation efforts in biological systems modeling, highlighting which parameters are crucial for the model’s behavior. The adopted research method leads to a feedback system that helps create a loop-based network for monitoring plant signaling pathways. Interdisciplinary collaboration is vital for addressing the challenge of understanding plant signaling pathways.

## Data Availability

All data codes related to this paper may be requested from the authors.
